# Editorial Comment to A case of adenocarcinoma of the rete testis with durable response to cisplatin‐based chemotherapy

**DOI:** 10.1002/iju5.12326

**Published:** 2021-06-21

**Authors:** Takeshi Yuasa

**Affiliations:** ^1^ Department of Urology Cancer Institute Hospital Japanese Foundation for Cancer Research Ariake Tokyo Japan

In spite of an unknown exact incidence, adenocarcinoma of the rete testis is extremely rare. Patients range in age from their 10s to their 90s, with a mean in the 50s and a peak incidence around the 70s’.^1^, ^2^ Most cases exhibit painless scrotal swelling as a clinical symptom, and half of patients have a history of hydrocele.^1^ Diagnosis is commonly made by histopathological analysis after high orchiectomy. One third of patients had metastases at presentation and half developed metastases after radical orchiectomy.^1^, ^2^ In nonmetastatic cases, after orchiectomy, although adjuvant radiotherapy for the retroperitoneal lymph node (RPLN) area does not appear to help, RPLN dissection may be a useful therapeutic modality.^1^ The value of adjuvant chemotherapy remains unknown.^1^, ^2^ The 3‐year survival rate of organ‐confined cases was nearly 90%,^1^, ^2^ and the prognosis of disseminated cases was reported to be poor.^1^

In this issue of *IJU Case Reports,* Owa *et al*. reported a case of adenocarcinoma of the rete testis in a 48‐year‐old man with Down syndrome.^3^ The patient had a 5.6 × 6.0 × 6.2 cm left testicular tumor within the hydrocele as well as multiple RPLN metastases.^3^ After left high orchiectomy, the diagnosis of adenocarcinoma of the rete testis was given based on the presence of *in situ* lesions in the rete testis and the exclusion of metastasis from other lesions.^3^ To treat the metastatic lesions, Owa *et al*. planned to administer four cycles of bleomycin, etoposide, and platinum (BEP) based on the standard treatment for intermediate/poor risk of metastatic testicular cancer. The patient developed severe side effects during the first cycle of BEP, however, and was subsequently switched to a reduced dose of EP therapy.^3^ The chemotherapy was terminated at six cycles without removal of the residual metastatic lesions.^3^ The metastatic lymph nodes remain nonenlarged 7 months after the chemotherapy was discontinued, and the patient is alive 20 months after discontinuing chemotherapy.^3^

In 1995, Sanchez‐Chapado *et al*. reviewed approximately 60 cases of adenocarcinoma of the rete testis.^1^ As some cases did not fulfill the diagnostic and histologic criteria, they summarized the clinical characteristics of 42 cases of this disease.^1^ Recently, Takeda *et al*. evaluated the demographic and clinical data of 51 cases.^2^ As described above, the prognosis of disseminated cases was poor, and 1‐ and 3‐year survival rates were reported to be 34% and 14%, respectively.^1^ The most common metastatic sites were the lung and lymph nodes followed by the skin, liver, and bone.^1^, ^2^ Due to its rarity, the recommended treatment strategies have not yet been established. When encountering a rare disease, we usually use the medical literature search engine, PubMed. As such, a description of the treatment is important despite the fact that the efficacy and value of chemotherapy have not yet been demonstrated.^1^, ^2^ I could understand that germ cell cancer‐oriented treatment is one of the most important options for metastatic adenocarcinoma of the rete testis.

## Conflict of interest

The author declares no conflict of interest.
